# Yves Buisson (1948-2025)

**DOI:** 10.48327/mtsi.v6i1.2026.827

**Published:** 2026-03-14

**Authors:** Jean-Paul BOUTIN

**Affiliations:** Vice-président de la SFMTSI, ancien titulaire de la Chaire d’épidémiologie et de prévention dans les armées Société francophone de médecine tropicale et santé internationale (ancienne SPE), Institut Pasteur, 25 Rue du Dr Roux, 75015 Paris, France

C’est l’élégance simple qui nous charme. Ovide (43 av. JC-18 ap. JC)

Science sans conscience n’est que ruine de l’âme. Rabelais (1483-1553)

**Figure 1:**
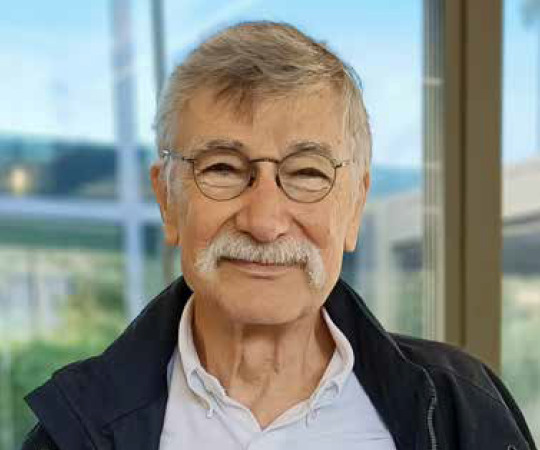
Yves Buisson en octobre 2021, lors du séminaire de la SFMTSI à Aix en Provence (crédit photo : Jean Jannin)

Yves Buisson s’est éteint le 26 novembre 2025 à Saint-Mandé, dans un de ces établissements du Service de santé des armées (SSA) qu’il avait tant servis. Connu, et mieux encore apprécié, dans tous les cénacles français les plus savants de la médecine, de la pharmacie, des sciences d’outre-mer, et dans toute la francophonie, il avait fait une entrée éclatante et remarquée sur la scène médiatique française en 2020 à l’occasion de l’hypercrise engendrée par la Covid-19. À cette occasion, il avait pleinement assumé la confiance de l’Académie nationale de médecine. Ballottée entre l’effroi bien naturel, les théories les plus hasardeuses et les diktats les moins étayés, la France découvrait un savant calme en toute circonstance, pédagogue et soucieux des humains. Un académicien n’hésitant pas à affirmer clairement le désaccord du Savoir face au Pouvoir. Il suffit pour cela de se souvenir de la polémique sur le port du masque. Et lorsqu’il fut avoué que le masque n’était point recommandable au motif qu’il n’y en avait plus en stock, Yves Buisson, président de la toute nouvelle Cellule de veille sur la Covid-19 de l’Académie, n’hésita pas à devenir le promoteur « rebelle » du masque-maison qu’il appela « masque grand public » tant que la puissance publique ne saurait subvenir aux besoins.

Mais pour en arriver à une telle aura scientifique et médiatique, souvenons-nous qu’un long apprentissage a façonné l’homme.

Yves Buisson est né le 2 février 1948 à Orléans, fils d’un cadre de la SNCF. Il est le deuxième de trois garçons qui tous deviendront militaires, un gendarme et deux médecins, et tous atteindront les sommets de leurs hiérarchies respectives.

Depuis l’entrée au lycée Henri IV à Paris en classe de 6^e^, il a parcouru avec son inséparable ami Bernard Lafont, futur professeur de psychiatrie au Val de Grâce, un trajet qui les fait intégrer l’École du Service de santé des armées (ESSA) de Lyon en 1965. Si sa scolarité y fut aussi brillante que linéaire, l’homme se distinguait déjà par quelques traits que les honneurs tendent à faire oublier. Yves était passionnément parachutiste. Dès la fin de la première année de médecine, en août 1966 il était breveté parachutiste à l’École des troupes aéroportées de Pau. Il ne s’agissait pas chez lui d’une simple qualification qui pourrait être utile dans la carrière, mais d’un goût affirmé, comme l’avait fait Alain son aîné en gendarmerie et comme le fera Philippe son benjamin lui aussi « santard » quelques années plus tard. Il pratiquera ce sport chaque année, à Pau comme à Lyon, et avec l’équipe de l’ESSA et son ami Dominique Baudon (futur professeur d’épidémiologie au Pharo) il devint en 1971 champion de France universitaire de parachutisme par équipe de 4 dans la discipline de précision d’atterrissage.

Quand Blandine entra dans sa vie, le parachutisme entra dans celle de Blandine ! Et comme premier poste en sortant d’école il choisit ainsi d’être médecin-adjoint au 6^e^ Régiment parachutiste d’infanterie de marine (RPIMa) à Mont-de-Marsan. Dans cette période universitaire, Yves Buisson se révèle aussi être un mélomane, et un chanteur au répertoire aussi large et fleuri que peut l’être celui des carabins français. Un répertoire que ni les grades, ni les titres, ni les positions les plus honorables et publiques ne lui feront oublier et qu’il fera partager sa vie durant pour peu que l’ambiance et l’entourage lui plaisent, avec cet œil pétillant d’humour et ce frissonnement de moustache caractéristiques de ses moments de détente.

Les trois années dans les Landes ne sont pas une sinécure, car en plus des obligations du service, Yves Buisson prépare le concours d’assistanat des hôpitaux des armées dans la discipline de biologie médicale, orientation qu’il avait découverte pendant son cursus lyonnais. Nommé assistant en 1976, il rejoint Paris et va pendant 4 ans parfaire sa formation de biologiste entre les hôpitaux militaires du Val de Grâce et Bégin, et l’Institut Pasteur. Il s’attache particulièrement à son patron au Val de Grâce, le Pr Henri-Michel Antoine avec lequel il nouera des liens affectueux, reconnaissants et respectueux sa vie durant.

Après son succès au concours de biologiste des hôpitaux des armées, il est nommé en 1980 au Sénégal, chef du laboratoire de microbiologie de l’Institut Pasteur de Dakar où il œuvre pendant 3 ans, et responsable du Centre national sénégalais des entérobactéries. C’est à l’occasion de ce séjour que débute réellement sa production scientifique. Il publie ainsi les résultats obtenus dans ce centre relatif aux entérobactéries mais aussi les données de la surveillance des urétrites et gonococcies au Sénégal et leur sensibilité aux antibiotiques, et encore une série de borrélioses ou des cas cliniques. Ce sont les débuts de sa collaboration avec notre Société de pathologie exotique (SPE) et son *Bulletin*.

En 1983, il est de retour au laboratoire de biologie clinique de l’Hôpital d’instruction des armées (HIA) du Val de Grâce, adjoint du Pr Pierre Saliou dorénavant chef de service. Il nouera avec ce dernier une amitié profonde qui les réunira bien au-delà de l’appartenance au Service de santé des armées, lorsque 4 ans plus tard Pierre quittera l’institution militaire pour une seconde carrière dans l’industrie des vaccins. Pendant leurs années communes, Yves Buisson est reçu dès 1985 à l’agrégation du Val de Grâce dans la discipline d’épidémiologie et prophylaxie dans l’armée de Terre. Ces années sont marquées par son appétence pour les hépatites virales et leur diagnostic. On a dit alors que dans les hôpitaux militaires franciliens, il régnait sur les marqueurs biologiques de l’hépatite B, alors même qu’il en facilita la maîtrise là où œuvraient des biologistes dont il appréciait le travail. C’est aussi l’époque des premiers travaux publiés dans quatre thématiques qui resteront des constantes de l’œuvre scientifique d’Yves Buisson pendant les deux décennies suivantes : l’antibiologie, la vaccinologie, les toxi-infections alimentaires collectives et les infections nosocomiales. Cet éclectisme, traditionnel et typique des praticiens hospitaliers militaires, paraît pourtant daté, voire critiqué dans le monde de la recherche biomédicale française. Il leur est souvent reproché par les tenants de l’hyperspécialisation. Il est pourtant le garant du concept d’honnête homme qu’incarnait Yves Buisson, selon lequel mieux vaut la recherche d’équilibre qu’une accumulation de savoirs, et comme l’affirmait Montaigne « *mieux vaut avoir la tête bien faite que bien pleine* ». Au soir de sa carrière scientifique, c’est bien cette vision d’ensemble des problèmes sanitaires d’origine infectieuse qui permettra à Yves Buisson d’affirmer ses convictions et de les promouvoir.

En 1987, après une escapade à Veyrier-du-Lac pour suivre le cours d’épidémiologie appliquée et de biostatistique des CDC (*Centers for Disease Control and Prevention*) d’Atlanta dans leur version francophone chère au Dr Charles Mérieux, Yves Buisson prend la chefferie du laboratoire de biologie clinique du Val de Grâce, et cela pour 12 ans, le temps de faire école. Car Yves est fondamentalement un passeur de savoir, et l’École d’application est encore le lieu parfait où les jeunes médecins militaires, formés dans les facultés françaises, découvrent les spécificités scientifiques de leur métier et la compétence pragmatique des enseignants du SSA. L’un d’eux témoigne près de 40 ans plus tard : « *à l’École d’application à Paris, en 1988, ce sont ses cours qui m’ont donné envie de choisir l’assistanat de biologie, et je ne suis pas le seul* ».

À l’hôpital, dans son laboratoire, Yves Buisson crée et entretient un climat de travail fait de rigueur dans la bonne humeur, où chaque jour apportait avec lui son lot de découvertes et d’émerveillement devant le monde microbien. Il avait hérité de cette conception de la biologie médicale que nos anciens dénommaient biologie clinique. Il affirmait haut et fort que les médecins biologistes ne devaient pas être des tâcherons cantonnés aux calibrages de machines et à l’utilisation d’algorithmes d’identification, mais des spécialistes au carrefour de la clinique, du laboratoire et de l’épidémiologie (entendue ici dans le sens de l’histoire naturelle des agents infectieux), et devant bien s’intéresser aux applications en matière vaccinales. L’année 1995 est celle de la grande reconnaissance de cet homme exceptionnel par la République, qui en juillet le fait chevalier de la Légion d’honneur. Ses pairs l’élisent Professeur titulaire de la Chaire d’épidémiologie et de prévention dans les armées en novembre, et la direction centrale du SSA le nomme Consultant national pour l’hygiène hospitalière dans les armées en décembre. La même année, la Direction générale de la santé (DGS) le nomme au Comité technique des vaccinations, alors qu’il est depuis deux ans déjà membre du Comité technique de lutte contre les infections nosocomiales.

**Figure 2:**
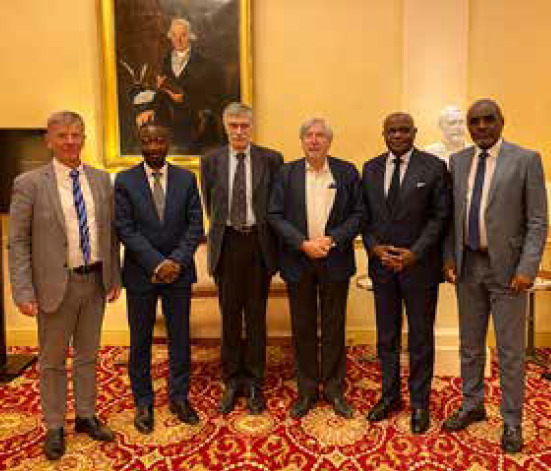
Les Pr Y. Buisson et P. Debré recevant les Pr J-R. Nzenze, J. Miloudja et R. Tchoua du Service de santé militaire et de l’Université des sciences et techniques de la santé du Gabon à l’Académie nationale de médecine le 1^er^ février 2023 (crédit photo : Dr. Alain Puidupin, à gauche)

Pendant ces 12 années de responsabilités au Val de Grâce, dans le SSA et auprès de la DGS, il initie, conduit, encourage et publie de nombreux travaux dans les domaines du diagnostic bactériologique, des résistances bactériennes aux antibiotiques, de l’infectiologie propre aux armées, des malades diarrhéiques – dont le choléra et les toxi-infections alimentaires collectives –, des infections sexuellement transmissibles et du sida, des infections nosocomiales, de la grippe, des hépatites virales de A à E, de la surveillance épidémiologique dans les armées, de la médecine des voyages et de la médecine tropicale, des nouveaux vaccins et des stratégies vaccinales, du risque d’agression biologique. Il propose et jette les bases d’une sérothèque du SSA, dirige la mission de la Bioforce militaire en Bolivie à l’occasion de l’émergence du choléra dans ce pays en 1992. Il investigue le risque de contamination des Forces françaises à Djibouti face aux épidémies récurrentes de choléra et de shigelloses qui sévissent dans la population et n’épargnent pas les militaires français. C’est aussi la période où il coordonne le Symposium international sur les virus hépatiques à transmission entérique tenu à Paris dont il publie les minutes (La Simmare, 1995), puis le rapport technique au Comité consultatif de santé des armées relatif aux diarrhées aiguës dans les armées (1998), puis encore l’ouvrage de synthèse relatif aux « Vaccinations dans les armées »^1^.

En 1999 la carrière d’Yves Buisson prend un tournant majeur. Il quitte le laboratoire pour des emplois de dirigeant d’établissements de recherche et d’enseignement. Dans ces derniers, il restera très proche de ses laboratoires et des étudiants. C’est d’abord, administrativement en position détachée du SSA, qu’il assure pendant deux ans la direction de l’Institut Pasteur du Cambodge à Phnom Penh. Parmi les travaux qu’il dirige ou inspire et qui ont été publiés, on n’est pas surpris de retrouver les thèmes constants de ses travaux : la sérologie du VIH, l’antibiorésistance, le virus de l’hépatite E et, étant toujours adepte de la biologie clinique, une réflexion sur la place de la chirurgie dans les hypertensions portales à *Schistosoma mekongi*. Quittant le Cambodge il laisse un souvenir et un respect qui se manifestent encore à l’occasion de son décès.

De retour à Paris en 2001, il est nommé délégué général au Réseau international des Instituts Pasteur et instituts associés à Paris. Cet emploi, où il succède à nombre de médecins militaires, aurait pu être un couronnement mais a été abrégé par un contexte de réorientation stratégique de l’Institut Pasteur.

En 2003, Yves Buisson est de retour au sein du Service de santé des armées. Il est chargé de mission auprès des organismes de recherche du SSA afin de coordonner l’action de ce service dans le domaine des risques biologiques, naturels comme provoqués. Dans cette période qui fait suite à la crise engendrée aux États-Unis par l’envoi postal de courriers contenant des souches de *Bacillus anthracis* (pour mémoire, 23 victimes dont 5 morts), et aux soupçons de présence de munitions biologiques au Moyen-Orient, le concept de risque B (pour biologique) repasse au-devant de la scène des risques pour les armées, comme pour la population française. La complexité du sujet nécessite que ce travail soit coordonné par une personnalité scientifiquement non discutable. C’est de cette expérience que sortira l’ouvrage « Les risques NRBC, savoir pour agir »^2^ dont l’objectif pour les auteurs était de « faire bénéficier de leur savoir-faire tous ceux et celles qui peuvent un jour être confrontés à un accident ou à un attentat radiologique, biologique ou chimique et qui souhaitent être capables de réagir utilement et rapidement ».

En 2004, Yves Buisson est promu officier de la Légion d’honneur et nommé directeur-adjoint de l’Institut de médecine tropicale du Service de santé des armées (IMTSSA), la célèbre École du Pharo à Marseille. Celle-ci est à la veille de son centenaire célébré en septembre 2005. Même si le projet de commémoration est programmé depuis plusieurs années, la dernière année est celle du *rush* permanent et de la diplomatie la plus exquise pour mener de front la tenue du 16^e^ Congrès international de médecine tropicale et du paludisme, du 4^e^ Congrès européen de médecine tropicale et de santé internationale, du 7^e^ Congrès de la SPE et des 12^e^ Actualités du Pharo, tout en ménageant les susceptibilités des politiques, des autorités militaires, des savants, des bailleurs, et sans que jamais le personnel ne se décourage. Les anciens présidents de la SPE se sont longtemps souvenus des affres engendrées par la tenue conjointe de ces évènements qui ont attiré plus de 1 500 personnes dans le parc du Pharo.

Dans la foulée, Yves Buisson prend la direction de l’établissement, en même temps qu’il reçoit ses 3 étoiles de médecin général inspecteur. Il y restera jusqu’au 14 décembre 2007. Pendant ses trois années à Marseille, il applique les mêmes méthodes de management qui avaient fait sa réputation au Val de Grâce : convictions, explications, sang-froid et sérénité, gant de velours, sourire et humour, exigence morale. Il y découvre aussi le projet local d’un futur infectiopôle qui verra le jour une décennie plus tard sous un autre nom. En quittant le Pharo, Yves Buisson quitte aussi les armées après 42 ans de service. Un nouveau défi principalement pédagogique lui est proposé. Il prend ainsi début 2008 la direction de l’Institut de la francophonie pour la médecine tropicale (IFMT) à Vientiane (Laos), organisme récemment créé par l’Agence universitaire de la francophonie en partenariat avec le gouvernement laotien, dans le but de proposer une formation qualifiante de niveau Master en santé publique et médecine tropicale, et de favoriser l’émergence de cadres dirigeants dans ces domaines en Asie du Sud-Est. Chaque année, une promotion d’une vingtaine de médecins et scientifiques nationaux ou du Sud-Est asiatique et de Madagascar y sont formés. Pour l’ancien directeur du Pharo, la boucle est bouclée. Comme nombre de grands anciens avant lui, que ce soit à Pey Yang en 1895, Hanoï en 1902, Shanghai en 1903, Phnom Penh en 1946, ou Vientiane en 1957, il dirige à son tour une école de cadres de santé en Asie tropicale. Nombre de ses anciens élèves œuvrent aujourd’hui au sein des Instituts Pasteur du Cambodge, du Laos et du Vietnam. Il est admis que son investissement a contribué à structurer la recherche en microbiologie et en santé publique dans la région.

En 2013, Yves et Blandine Buisson rentrent en France pour ce qu’il est convenu d’appeler la retraite.

Auteur d’environ 270 articles scientifiques, 41 chapitres d’ouvrages, 26 rapports scientifiques et techniques, et coordonnateur de plusieurs livres, Yves est alors une personnalité reconnue du monde scientifique et médical français.

Dès 2002, il avait été élu membre correspondant de l’Académie nationale de médecine. Il en devint membre titulaire en 2007 dans la 4^e^ division « Santé publique ». Yves Buisson œuvrait au sein de la Commission VI « Une seule santé humaine et animale, Maladies infectieuses, Vaccins » qu’il finira par présider. Son implication a été décisive concernant les vaccinations, sujet qui lui était particulièrement cher. Il y a travaillé en faveur de la vaccination contre les hépatites et contre les infections respiratoires, et a été un moteur dans la rédaction d’importants rapports et communiqués sur les vaccinations des seniors et des femmes enceintes.

À partir du 19 mars 2020, il préside la cellule de veille nouvellement créée et dénommée « Épidémie de Covid-19 » de l’Académie dont il fût l’âme. C’est dans cette dernière responsabilité qu’il va donner toute la mesure de son autorité scientifique, de ses qualités de pédagogue et de communicant, et montrer sa force de conviction. Il avait un sens remarquable de la formule pour forger des titres percutants et un esprit synthétique lui permettant la concision, ce qui valorisait considérablement la diffusion des informations. C’est ainsi que l’Académie de médecine a pu jouer un rôle majeur en France dans la lutte contre la pandémie de Covid-19 grâce à une communication fiable, en accompagnant les grandes orientations et en luttant contre les *fake news*. Dès le 22 mars 2020, l’Académie de médecine prit position sur l’importance des masques, qui était un sujet de controverse, quitte à provoquer en clamant « *Aux masques citoyens* ». Sous son impulsion, pour la seule année 2020, cette cellule a produit près de 110 communiqués concernant la Covid-19. Ceux-ci informaient les pouvoirs publics, les médias et le grand public sur les développements de la pandémie et les précautions pour se protéger. Ils concernaient toutes les questions que se posait le grand public : les tests virologiques, la vaccination, le confinement, les problèmes liés aux personnes fragiles, aux aînés en EHPAD, aux personnes défavorisées, au Covid-19 long et à ses séquelles, à l’impact en Afrique et dans le monde, aux mutations du virus... Yves Buisson a joué un rôle majeur dans cette crise du fait de ses qualités de rassembleur, obtenant facilement des consensus. Un site spécialisé a établi la statistique des personnalités étant intervenues sur le sujet de la Covid-19 en 2020 sur les médias français. Au cours du premier semestre, Yves Buisson était la 36^e^ personnalité la plus présente sur les médias nationaux (derrière 12 hauts responsables politiques) avec 14 interventions. Au second semestre, Yves Buisson était la 9^e^ personnalité la plus présente sur les médias nationaux (derrière 4 hauts responsables politiques) avec 29 interventions. La bonne communication ne trompe pas !

Yves Buisson était membre associé de l’Académie nationale de pharmacie et venait d’être élu membre titulaire de la 4^e^ section de l’Académie des sciences d’outre-mer sur le siège de son ami Pierre Saliou. Membre de 13 sociétés savantes, Yves était resté très proche du Groupe d’intervention en santé publique et épidémiologie (GISPE), lequel continue d’organiser chaque année le congrès de médecine et santé tropicale dit « Les Actualités du Pharo » créé en 1994 par les enseignants de l’école qu’il avait lui-même fini par diriger. Le 8 octobre 2025, malgré l’avancée de la maladie qui devait l’emporter quelques semaines plus tard, il avait tenu à honorer ses engagements en y prononçant une brillante conférence intitulée « Panorama des vaccins pour les populations des zones tropicales ».

Il était membre de la Société de pathologie exotique (SPE) depuis 1983, dont il fut le secrétaire général puis le président de 2015 à 2018. Sous son mandat et son impulsion, la SPE a organisé au Vietnam son 10^e^ Congrès international à l’université de Médecine et de Pharmacie de Haiphong (UMPH) du 8 au 10 novembre 2017 sur le thème de l’accès à la chirurgie en zones tropicales. L’UMPH avait été choisie parce qu’elle entretenait une importante collaboration avec les universités françaises, et parce que son président, le Pr Pham Van Thuc, venait d’être élu membre correspondant étranger de l’Académie de médecine, tandis qu’Yves Buisson était docteur *honoris causa* de l’université de Médecine et de Pharmacie de Haiphong. Les partenaires vietnamiens avaient souhaité axer ce congrès sur le thème de l’accès à la chirurgie en zones tropicales et des actualités en oncologie. Il s’agissait donc d’un congrès mixte avec la Conférence nationale vietnamienne de cancérologie et la Fondation de l’Académie de médecine en était partenaire. Cet évènement avait été unanimement considéré comme un succès en francophonie.

Depuis lors, Yves Buisson avait toujours gardé des liens très étroits avec la SPE, membre actif de son conseil scientifique et acteur de ses Journées scientifiques et des Actualités du Pharo, dont notre société est dorénavant co-organisatrice.

Yves Buisson était officier de la Légion d’honneur, officier de l’ordre national du Mérite, titulaire de la médaille d’honneur du Service de santé des armées et de plusieurs distinctions laotiennes et vietnamiennes. Pour ses travaux scientifiques, il était lauréat du Prix Noury-Lemarié attribué en 1984 par la SPE pour ses travaux sur *Neisseria gonorrhoeae* en pays tropicaux, et il fut le premier lauréat du très exceptionnel échelon Or de la médaille du Service de santé des armées pour travaux scientifiques.

**Figure 3:**
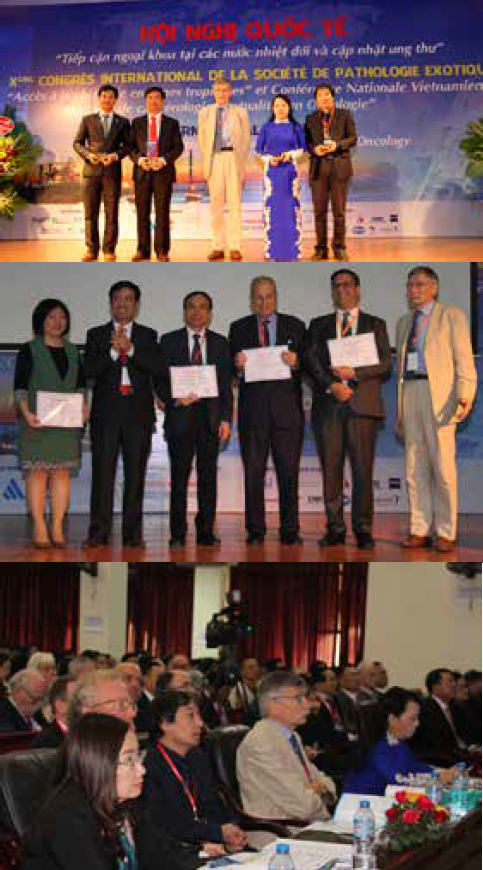
10^e^ Congrès international à l’université de Médecine et de Pharmacie de Haiphong, Vietnam (Crédit photo : UMPH)

## Épilogue

Yves et Blandine Buisson formaient un couple merveilleux et fusionnel, que la vie n’a cependant pas épargné. C’est ensemble qu’ils ont construit ce parcours emblématique du savoir et de l’élégance française. Ils resteront pour tous un exemple de vie.

Tous ceux et celles qui ont travaillé auprès d’Yves se souviennent de ces moments de détente qui lui ressemblaient, où ses yeux pétillaient et sa moustache frissonnait, dont le traditionnel accueil au troisième jeudi de novembre du Beaujolais nouveau. En 2026, pour tous ceux qui ont tant apprécié Yves Buisson le breuvage aura un goût amer.

L’auteur tient à remercier tous les camarades et confrères qui, ayant connu et travaillé avec Yves Buisson à des étapes différentes de leurs parcours, ont accepté de témoigner pour l’écriture de ce texte. Ils se reconnaîtront. Merci Bernard, François, Dominique, Jean, Patrick, Vincent, Marc, et encore Marc, Jean-Louis, encore Jean et Jean-Philippe.

## Footnotes

1 Buisson Y. Vaccination dans les armées (1999). Addim éd. Paris. 234 p. ISBN 2-907341-81-2

2 Buisson Y. et col. Les risques NRBC, savoir pour agir (2004). Xavier Montauban éd. Montrouge. 300 p. et 1 CD. ISBN : 978-2-914990-01-1
