# Supplementary material for: Yves Buisson (1948-2025)
Source: Med Trop Sante Int. 2026 Mar 14;6(1):mtsi.v6i1.2026.827. [Article in French] doi: 10.48327/mtsi.v6i1.2026.827 (PMC13216908; doi:10.48327/mtsi.v6i1.2026.827)
Supplement: Related texts

# Research article: Yves Buisson (1948-2025)

It is simple elegance that charms us. Ovid (43 BC–18 AD)

Science without conscience is but the ruin of the soul. Rabelais (1483-1553)

**Figure 1:** Yves Buisson in October 2021, during the SFMTSI seminar in Aix-en-Provence (photo credit: Jean Jannin)

Yves Buisson passed away on November 26, 2025, in Saint-Mandé at a facility of the *Service de santé des armées* (SSA, Joint Health Service), which he served so well. He was well known and highly regarded in the most distinguished French medical, pharmaceutical, and overseas scientific circles, as well as throughout the French-speaking world. He made a striking and remarkable entrance onto the French media scene in 2020 during the hypercrisis caused by the Covid-19 pandemic. At that time, he fully lived up to the trust placed in him by the *Académie nationale de médecine*. Torn between natural fear, hazardous theories, and unsubstantiated dictates, France discovered a scholar who remained calm in all circumstances, pedagogical and was concerned for humanity. He was an Academician who did not hesitate to assert the disagreement of Knowledge with the Power. One need only recall the controversy over wearing face masks. When it was admitted that masks were not recommended due to a shortage, Yves Buisson, president of the Academy’s newly created Covid-19 Monitoring Unit, became the “rebel” promoter of homemade masks, calling them “general public masks,” as long as public authorities were unable to meet demand.

However, to achieve such scientific and media acclaim, it is important to remember that a long apprenticeship shaped the man.

Yves Buisson was born on February 2, 1948, in Orléans to a Railway compagny executive. He is the second of three boys, all of whom became military officers: one was a police officer, and two were doctors. All of them rose to the top of their respective hierarchies.

He and his inseparable friend Bernard Lafont, who would become a professor of psychiatry at the Val de Grâce, entered the Lycée Henri IV in Paris in the 6^th^ grade (France). They followed a path that led them to enroll in the École du *Service de Santé des Armées* (Military Health Academy, ESSA) in Lyon in 1965. Although his academic career was brilliant and linear, he stood out for a few traits that honors tend to make us forget. Yves was a passionate parachutist. At the end of his first year of medical school, in August 1966, he received his parachutist certification from the Airborne Troops School in Pau. To him, it was more than just a useful qualification; it was a strong passion, as it had been for his older brother Alain in the gendarmerie and as it would be for his younger brother Philippe, also a medical student, a few years later. He practiced this sport every year in Pau and Lyon with the ESSA team. With his friend Dominique Baudon, a future professor of epidemiology at Le Pharo, he became the French university champion in the 4-person team precision landing discipline in 1971.

When Blandine came into his life, skydiving came into hers! His first job after school was as an assistant doctor with the 6^th^ Parachute Infantry Marine Regiment (RPIMa) in Mont-de-Marsan. During his university years, Yves Buisson revealed himself to be a music lover and singer with a wide and colorful repertoire, like that of French medical students. Neither rank, title, nor the most honorable and public positions could make him forget it, and he shared it throughout his life as long as the atmosphere and company suited him. He had a twinkle of humor in his eye and a characteristic twitch of his mustache when he was relaxing.

The three years in the Landes (South-West France) were no picnic. In addition to his service obligations, Yves Buisson prepared for the army hospital assistant exam in medical biology, a specialty he discovered during his studies in Lyon. After being appointed assistant in 1976, he moved to Paris, where he spent four years refining his training as a biologist at the Val de Grâce and Bégin military hospitals, as well as the Pasteur Institute. He became particularly attached to his boss at Val de Grâce, Professor Henri-Michel Antoine. They formed a close bond based on gratitude and respect that lasted throughout their lives.

After passing the Army Hospital Biologist Exam in 1980, he was appointed head of the microbiology laboratory at the Pasteur Institute in Dakar, Senegal, where he worked for three years. He also became head of the Senegalese National Center for enterobacteria. It was during this time that his scientific work truly began. He published the results obtained at the center on enterobacteria, as well as data on the surveillance of urethritis and gonococcal infections in Senegal and their sensitivity to antibiotics and a series of cases of borreliosis. This marked the beginning of his collaboration with the *Société de pathologie exotique* (SPE) and its *Bulletin*.

In 1983, he returned to the clinical biology laboratory at the Val de Grâce as an assistant to Professor Pierre Saliou, who was now the head of the department. The two formed a deep friendship that continued long after they left the Armed Forces Health Service. Four years later, Pierre left the military to pursue a second career in the vaccine industry. During their years together, Buisson was accepted into the Val de Grâce teaching competiton in the discipline of epidemiology and prophylaxis in the army in 1985. These years were marked by his interest in viral hepatitis and its diagnosis. At the time, it was said that he dominated the field of hepatitis B biological markers in military hospitals in the Paris region, although he facilitated biologists’ work, which he appreciated. It was also during this period that the first works were published on four themes that would remain constants in Buisson’s scientific work for the next two decades: antibiotics, vaccinology, food poisoning outbreaks, and nosocomial infections. This eclecticism is traditional among military hospital practitioners but seems outdated and is criticized in the world of French biomedical research. Advocates of hyperspecialization often criticize it. However, this eclecticism is the guarantor of the concept of the honest man, as embodied by Yves Buisson. According to this concept, it is better to seek balance than to accumulate knowledge. As Montaigne said, “It is better to have a well-formed mind than a well-filled one.” This comprehensive view of infectious health problems enabled Yves Buisson to assert and promote his convictions at the end of his scientific career.

In 1987, after a trip to Veyrier-du-Lac to attend the French-language version of the CDC (Centers for Disease Control and Prevention, Atlanta) course in applied epidemiology and biostatistics, Yves Buisson took over as head of the clinical biology laboratory at the Val de Grâce. During his 12-year tenure, he trained a whole new generation. Yves is fundamentally a transmitter of knowledge, and the École d’Application (Joint Medical Academy) remains the perfect place for young military doctors trained in French universities to learn the scientific specifics of their profession and develop the practical skills of the SSA’s instructors. Nearly 40 years later, one of his students testified: “*It was his classes at the École d’Application in Paris in 1988 that made me want to choose biology as my specialty, and I’m not the only one*.”

In his laboratory at the hospital, Yves Buisson fostered an environment characterized by rigor and good humor where each day brought new discoveries and a sense of wonder about the microbial world. He inherited this conception of medical biology from our elders, who called it clinical biology. He asserted that medical biologists should be specialists at the crossroads of clinical practice, the laboratory, and epidemiology, taking a keen interest in vaccine applications, rather than mere laborers confined to calibrating machines and using identification algorithms.

In 1995, this exceptional man received significant recognition from the Republic. In July, he was made a Knight of the Legion of Honor. In November, his peers elected him full professor of epidemiology and prevention in the armed forces, and in December, the central management of the SSA appointed him national consultant for hospital hygiene in the armed forces. The General Directorate of Health (DGS, civilian) also appointed him to the Technical Committee on Vaccinations that same year, although he had already been a member of the Technical Committee Against Nosocomial Infections for two years.

During his twelve years of service at Val de Grâce, within the SSA, and with the DGS, he initiated, led, and encouraged numerous projects in the fields of bacteriological diagnosis, bacterial resistance to antibiotics, infectious diseases specific to the armed forces, diarrhea-causing diseases (including cholera and food poisoning), sexually transmitted infections, AIDS, nosocomial infections, influenza, viral hepatitis (types A-E), epidemiological surveillance in the armed forces, travel medicine, tropical medicine, new vaccines, and vaccination strategies. He also addressed the risk of biological attack. He proposed the creation of an SSA serum bank and led the military Bioforce mission in Bolivia when a cholera outbreak occurred in the country in 1992. He investigated the risk of French force contamination in Djibouti amid recurrent cholera and shigellosis epidemics ravaging the population and sparing no one, including French soldiers. During this period, he also coordinated the International Symposium on Enterically Transmitted Hepatic Viruses in Paris and published the minutes (La Simmare, 1995). He then published the technical report on acute diarrhea in the armed forces to the Armed Forces Health Advisory Committee (1998) and finally the summary book on “Vaccinations in the Armed Forces^1^”.

**Figure 2:** Prof. Y. Buisson and P. Debré welcoming Prof. J-R. Nzenze, J. Miloudja, and R. Tchoua from the Military Health Service and the University of Health Sciences and Technology of Gabon to the National Academy of Medicine on February 1, 2023 (photo credit: Dr. Alain Puidupin, left)

In 1999, Yves Buisson’s career took a major turn. He left the laboratory to become a director of research and teaching establishments. In these roles, he remained close to his laboratories and students. First, he spent two years as director of the Pasteur Institute of Cambodia in Phnom Penh on secondment from the SSA. Among the works he directed or inspired, the constant themes of his work are evident: HIV serology, antibiotic resistance, and hepatitis E virus. As he was always a fan of clinical biology, he also wrote about the role of surgery in portal hypertension in *Schistosoma mekongi*. Upon leaving Cambodia, he left behind memories and respect that are still evident today. Upon returning to Paris in 2001, he was appointed General Delegate of the International Network of Pasteur Institutes and Associated Institutes. This position in which he succeeds a number of military doctor, could have been the crowning achievement of his career, it was cut short by a strategic reorientation of the Pasteur Institute. In 2003, he returned to the Armed Forces Health Service. He was appointed special advisor to the SSA’s research organizations, where he coordinated the service’s activities in the field of biological risks, both natural and man-made. Following the crisis in the United States involving letters containing *Bacillus anthracis* (23 victims, including five deaths) and the suspicion of biological weapons in the Middle East, biological risk became a primary concern for the armed forces and the French population. The complexity of the subject required coordination by a scientifically credible figure. This experience led to the publication of the book “*Les Risques NRBC: Savoir Pour Agir^2^”* (CBRN Risks: Knowledge for Action), whose authors aimed to “share their expertise with all those who may one day be confronted with a radiological, biological, or chemical accident or attack and wish to respond effectively and quickly.”

In 2004, Yves Buisson was promoted to Officer of the Legion of Honor and was appointed deputy director of the *Institut de médecine tropicale du Service de santé des armées* (IMTSSA) at the renowned École du Pharo in Marseille. The institute was on the eve of its centenary, which was celebrated in September 2005. Although the commemoration project had been in progress for several years, the final year was marked by a constant sense of urgency and delicate diplomacy as the institute managed the 16th International Congress of Tropical Medicine and Malaria, the 4th European Congress of Tropical Medicine and International Health, the 7^th^ SPE Congress, and the 12^th^ *Actualités du Pharo*. Throughout this time, the staff successfully navigated the sensitivities of politicians, military authorities, scientists, and donors without ever becoming discouraged. Former SPE presidents still remember the turmoil caused by holding these events jointly, which attracted over 1,500 people to Pharo Park.

Following this, Buisson took over as director of the institution while receiving his stars as a Major general . He remained there until December 14, 2007. During his three years in Marseille, he applied the same management methods that had earned him a reputation at Val de Grâce: conviction, explanation, composure, serenity, a velvet glove, a smile, humor, and moral standards. He also discovered the local project for a future infectious disease center, which would be established a decade later under a different name. When he left Le Pharo, Yves Buisson also left the army after 42 years of service. He was offered a new challenge, mainly in the field of education. In early 2008, he took over as director of the *Institut de la Francophonie pour la Médecine Tropicale* (IFMT) in Vientiane, Laos, an organization recently created by the *Agence universitaire de la francophonie* in partnership with the Laotian government, with the aim of offering a master’s degree in public health and tropical medicine and promoting the emergence of senior executives in these fields in Southeast Asia. Each year, a class of around 20 doctors and scientists from Laos, Southeast Asia, and Madagascar are trained there. For the former director of Pharo, this brings him full circle. Like many of his predecessors before him, whether in Pey Yang in 1895, Hanoi in 1902, Shanghai in 1903, Phnom Penh in 1946, or Vientiane in 1957, he in turn heads a school for health executives in tropical Asia. Many of his former students now work at the Pasteur Institutes in Cambodia, Laos, and Vietnam. It is widely acknowledged that his dedication has helped to structure microbiology and public health research in the region.

In 2013, Yves and Blandine Buisson returned to France for what is commonly referred to as retirement.

Author of some 270 scientific articles, 41 book chapters, 26 scientific and technical reports, and coordinator of several books, Yves was by then a recognized figure in the French scientific and medical world.

He was elected a corresponding member of the *Académie nationale de médecine* in 2002. In 2007, he became a full member of the 4^th^ division, “Public Health.” He worked within Commission VI, “One Health, Infectious Diseases, Vaccines,” eventually chairing it. He played a decisive role in the field of vaccinations, a subject particularly close to his heart. He promoted vaccination against hepatitis and respiratory infections and played a key role in drafting important reports and communiqués on vaccinations for seniors and pregnant women.

Starting on March 19, 2020, he chaired the Academy’s newly created monitoring unit, “Covid-19 Epidemic,” which he spearheaded. In this role, he demonstrated the full extent of his scientific authority, teaching skills, and strength of conviction. He had a remarkable knack for crafting powerful headlines, and his ability to be concise greatly enhanced the dissemination of information. This enabled the Academy of Medicine to play a significant role in France’s fight against the pandemic by providing reliable information, supporting key guidelines, and combating misinformation. On March 22, 2020, the Academy of Medicine took a stand on the importance of masks, a controversial topic, proclaiming “*Aux masques, citoyens!*” (Citizens, wear your masks! A barely veiled reference to the French national anthem). Under his leadership, the unit produced nearly 110 press releases concerning the pandemic in 2020 alone. These releases informed public authorities, the media, and the general public about developments in the pandemic and precautions they could take to protect themselves. The releases covered all the questions the public had: virological testing, vaccination, lockdown, issues relating to vulnerable people, the elderly in nursing homes, the disadvantaged, long-term effects of the virus, its impact in Africa and around the world, and mutations of the virus. Yves Buisson played a significant role in this crisis thanks to his ability to unify and reach consensus quickly. A specialized website compiled statistics on the individuals who spoke about the subject of Covid-19 in the French media in 2020. In the first half of the year, Yves Buisson was the 36^th^ most prominent figure in the national media, behind 12 senior politicians, with 14 appearances. In the second half of the year, he was the ninth most prominent figure in the national media, behind four senior politicians, with 29 appearances. Good communication does not deceive!

Yves Buisson, an associate member of the National Academy of Pharmacy, had just been elected a full member of the Fourth Section of the Overseas Academy of Sciences, succeeding his friend Pierre Saliou. Yves was a member of thirteen learned societies and remained very close to the *Groupe d’intervention en santé publique et épidémiologie* (GISPE). GISPE continues to organize the annual tropical medicine and health conference, “*Les Actualités du Pharo*,” created in 1994 by the teachers of the school that Yves later directed. Despite the progression of the disease that would take his life a few weeks later, on October 8, 2025, he insisted on honoring his commitment to give a brilliant lecture entitled “Panorama of Vaccines for Populations in Tropical Areas.”

He had been a member of the *Société de pathologie exotique* (SPE) since 1983 and served as secretary general and then president from 2015 to 2018. Under his leadership, the SPE organized its 10^th^ International Congress at the Hai Phong Medical University (HPMU) in Vietnam from November 8 to 10, 2017. The theme was access to surgery in tropical areas. The HPMU was chosen because of its strong collaboration with French universities, and because its president, Prof. Pham Van Thuc, had just been elected a foreign corresponding member of the Academy of Medicine. Meanwhile, Yves Buisson was an honorary doctor of the University of Medicine and Pharmacy of Haiphong. The Vietnamese partners wanted to focus the congress on access to surgery in tropical areas and current developments in oncology. Therefore, it was a joint congress with the Vietnamese National Cancer Conference, and the Foundation of the Academy of Medicine was a partner. This event was considered a resounding success in the French-speaking world.

Since then, Yves Buisson has maintained close ties with the SPE as an active member of its Scientific Council, participating in its Scientific Days and *Actualités du Pharo*, which our company now co-organizes.

He was an Officer of the Legion of Honor and of the National Order of Merit, a holder of the Medal of Honor of the Armed Forces Health Service, and a recipient of several Laotian and Vietnamese distinctions. He was awarded the Noury-Lemarié Prize by the SPE in 1984 for his work on *Neisseria gonorrhoeae* in tropical countries. He was also the first recipient of the highly exceptional Gold Medal of the Armed Forces Health Service for scientific work.

**Figure 3:** 10^th^ International Congress at the Hai Phong Medical University (HPMU) in Vietnam (photo credit: HPMU)

## Epilogue

Yves and Blandine Buisson were a wonderful, close-knit couple, but life did not spare them. Together, they built a career that epitomized French knowledge and elegance. They will remain an example of how to live life for everyone.

Those who worked with Yves remember the moments when he relaxed, his eyes sparkling and his mustache quivering. One such moment was the traditional welcome on the third Thursday of November for the Beaujolais Nouveau. In 2026, for those who appreciated Yves Buisson, the drink will have a bitter taste.

The author would like to thank his comrades and colleagues who knew and worked with Yves Buisson at different stages of their careers and who agreed to contribute to this text. They will recognize themselves. Thank you, Bernard, François, Dominique, Jean, Patrick, Vincent, Marc, Marc again, Jean-Louis, Jean again and Jean-Philippe.

1Buisson Y. Vaccination dans les armées (1999). Addim éd. Paris. 234 p. ISBN 2-907341-81-22Buisson Y. *et al.* Les risques NRBC, savoir pour agir (2004). Xavier Montauban éd. Montrouge. 300 p. et 1 CD. ISBN : 978-2-914990-01-1
